# Rates of and factors associated with exclusive and any breastfeeding at six months in Canada: an analysis of population-based cross-sectional data

**DOI:** 10.1186/s12884-023-05382-2

**Published:** 2023-01-23

**Authors:** Christina Ricci, Victoria Otterman, Terri-Lyn Bennett, Stephanie Metcalfe, Elizabeth Darling, Sonia Semenic, Susie Dzakpasu, for the Canadian Perinatal Surveillance System

**Affiliations:** 1 Lifespan Chronic Disease and Conditions Division, Public Health Agency of Canada, Ottawa, ON Canada; 2 Department of Obstetrics & Gynecology, McMaster University, Hamilton, ON Canada; 3 Ingram School of Nursing, McGill University, Montreal, QC Canada

**Keywords:** Breastfeeding, Infants, Associations, Canada

## Abstract

**Background:**

Breastfeeding has many health, economic and environmental benefits for both the infant and pregnant individual. Due to these benefits, the World Health Organization and Health Canada recommend exclusive breastfeeding for the first six months of life. The purpose of this study is to examine the prevalence of exclusive and any breastfeeding in Canada for at least six months, and factors associated with breastfeeding cessation prior to six months.

**Methods:**

We performed a secondary analysis of breastfeeding-related questions asked on the cross-sectional 2017–2018 Canadian Community Health Survey. Our sample comprised 5,392 females aged 15–55 who had given birth in the five years preceding the survey. Descriptive statistics were carried out to assess the proportion of females exclusively breastfeeding and doing any breastfeeding for at least six months by demographic and behavioural factors. We also assessed, by baby’s age, trends in the introduction of solids and liquids, breastfeeding cessation and the reasons females stopped breastfeeding. Multivariate log binominal regression was used to examine the association between breastfeeding at six months and selected maternal characteristics hypothesized a priori to be associated with breastfeeding behaviour.

**Results:**

Overall, for at least six months, 35.6% (95% confidence interval (CI): 33.3%-37.8%) of females breastfed exclusively and 62.2% (95% CI: 60.0%-64.4%) did any breastfeeding. The largest decline in exclusive breastfeeding occurred in the first month. Factors most strongly associated with breastfeeding for at least six months were having a bachelor’s or higher degree, having a normal body mass index, being married and daily co-sleeping. Insufficient milk supply was given as the most common reason for breastfeeding cessation irrespective of when females stopped breastfeeding.

**Conclusion:**

Six-month exclusive breastfeeding rates in Canada remain below targets set by the World Health Assembly. Continued efforts, including investment in monitoring of breastfeeding rates, are needed to promote and support exclusive breastfeeding, especially among females vulnerable to early cessation**.**

**Supplementary Information:**

The online version contains supplementary material available at 10.1186/s12884-023-05382-2.

## Introduction

Breastfeeding has established health, economic and environmental benefits, including improved cognitive development among infants and lowering the risk of infections, diabetes and cancers in children and females [1–3]. In recognition of such benefits, the World Health Organization (WHO) and Health Canada recommend exclusive breastfeeding for the first six months of life [4], which is defined as receiving breast milk (including expressed breast milk) and no other liquid (including water) or solid foods with the exception of nutritional supplements and medications [5].

Previous studies have shown that many females in Canada do not meet the WHO/Health Canada recommendation. Data from the 2006–2007 Canadian Maternity Experience Survey indicated that only 14.4% of females exclusively breastfed for at least six months [6]; and that 25% of women who initiated breastfeeding added liquids other than breastmilk to their child’s diet within two weeks of delivery [7]. By 2011–2012, six-month exclusive breastfeeding rates had increased, but remained low at 26% [8]. Of additional concern is that rates tend to vary significantly across sociodemographic groups signalling that breastfeeding behaviour is influenced by numerous social determinants of health [9, 10].

We examined six-month exclusive breastfeeding rates and factors associated with breastfeeding duration. Any breastfeeding was also examined. These analyses will update published Canadian breastfeeding statistics by six years using the most complete and representative data available and further inform health professionals about Canadian breastfeeding patterns.

## Methods

### Data

We used data from the 2017 and 2018 CCHS, which is a national cross-sectional survey conducted by Statistics Canada that includes questions about breastfeeding and other maternal characteristics. This survey provides the most current and nationally representative data on breastfeeding, with a response rate of 60.7%. Breastfeeding questions were asked of females aged 15–55 who gave birth in the five years preceding the survey, about their youngest child. In the case of a multiple birth, one of the children was picked at random. This corresponded to females who gave birth between 2012 and 2018. There were 5,392 females who met this inclusion criterion, representing 5.0% of 2017–2018 CCHS respondents. From this cohort, we excluded 148 individuals (2.7% of cohort) with missing information on whether they breastfed. We further excluded 399 females (7.4%) with children less than six months old who were still breastfeeding at the time of the survey, as they would not have had the opportunity to breastfeed for six months. The remaining 4,845 females (weighted to 1,471,316 females representative of the Canadian population) constituted the cohort used for the “any breastfeeding” analysis. An additional 104 females (1.9%) were excluded from the “exclusive breastfeeding” analysis, as they were missing information on the timing of introduction of liquids and/or solids**.** These analyses were therefore based on 4,741 females (weighted to 1,443,068 females representative of the Canadian population)**.** Not all people who breast/chestfeed identify as ‘female’; however we use this term because it corresponds to the phrasing used in the CCHS. Further details on the CCHS can be found on the Statistics Canada website [11].

### Measures

The main outcome of the study was exclusive breastfeeding for at least six months. Exclusive breastfeeding duration was derived from a question on whether the respondent breastfed (Yes/No) and questions about the age of the infant when other liquids and/or solids were introduced into the infant’s diet and the length of the breastfeeding. Any breastfeeding duration was derived from the question on whether the respondent breastfed (Yes/No) and a question on how long they breastfed. Exclusive breastfeeding duration was measured in months corresponding to the age of the baby when other liquids or solids were added to the baby’s diet. Any breastfeeding duration was measured in months corresponding to the age of the baby when any breastfeeding stopped.

The following maternal characteristics were determined a priori to have a potential association with breastfeeding duration based on previous literature [3–5, 8–10]: maternal age, province or territory of residence, educational attainment, household income, population group, immigrant status, pre-pregnancy body mass index (BMI), rural/urban residence, smoking status during the last three months of pregnancy, co-sleeping with baby, marital status and perceived mental health. The CCHS defined co-sleeping as the baby and parent(s) sharing the same bed. The analytical categories used for all measures are indicated in Table 1. We subsequently excluded household income from our analysis because it was not collected for respondents living in Canada’s three territories. A sensitivity analysis restricted to females living in the provinces found that results were not significantly impacted by this exclusion. Due to sample size limitations, First Nations, Metis and Inuit females were grouped as Indigenous. With the exception of pre-pregnancy BMI, smoking status during pregnancy, and co-sleeping with baby, all data reflect the respondent’s condition on the day of the survey which could be different from the respondent’s condition in the six months following the index birth.

**Table 1:** Exclusive and any breastfeeding rates for at least six months, by maternal characteristics, 2017–2018 Canadian Community Health Survey

	Exclusive breastfeeding for at least 6 months^a^		Any breastfeeding for at least 6 months^a^	
	Unweighted N	Weighted rate	Unweighted N	Weighted rate
Canada	1617	35.6% (33.3%-37.8%)	2885	62.2% (60.0%-64.4%)
Age				
15–19^b^				
20–24	69	24.6% (17.2%-32.0%)^c^	117	36.6% (28.9%-44.3%)
25–29	276	28.3% (23.8%-32.8%)	524	56.6% (51.9%-61.4%)
30–34	569	34.8% (31.2%-38.3%)	1039	64.3% (60.8%-67.7%)
35–39	487	40.5% (35.8%-45.2%)	825	65.8% (61.3%-70.3%)
40–44	189	41.6% (34.4%-48.7%)	327	69.7% (63.3%-76.1%)
45–55	22	36.1% (13.4%-58.7%)^c^	41	47.6% (24.0%-71.1%)^c^
Region				
Newfoundland	28	21.8% (13.3%-30.3%)^c^	45	46.6% (33.4%-59.8%)
Prince Edward Island	18	27.0% (15.0%-39.1%)^c^	35	54.9% (40.6%-69.2%)
Nova Scotia	48	27.7% (19.2%-36.3%)^c^	85	50.5% (40.9%-60.1%)
New Brunswick	33	39.5% (27.1%-51.8%)^c^	58	57.1% (45.7%-68.6%)
Quebec	246	26.1% (22.0%-30.2%)	542	54.3% (50.0%-58.5%)
Ontario	470	37.4% (32.9%-41.8%)	815	64.6% (60.3%-69.0%)
Manitoba	109	42.9% (34.7%-51.1%)	184	65.6% (57.7%-73.5%)
Saskatchewan	96	40.7% (31.4%-50.1%)	147	60.5% (51.8%-69.3%)
Alberta	264	33.5% (28.6%-38.4%)	479	62.4% (57.1%-67.6%)
British Columbia	247	51.3% (44.6%-58.0%)	396	76.0% (71.1%-80.8%)
Yukon	23	65.2% (48.2%-82.3%)	38	90.1% (78.0%-100%)
Northwest Territories	19	33.1% (19.1%-47.1%)^c^	33	53.5% (38.3%-68.7%)
Nunavut		^b^	28	33.9% (17.6%-50.2%)^c^
Education – Respondent				
Less than high school	82	25.6% (18.0%-33.2%)^c^	159	46.8% (38.5%-55.0%)
High school graduate	259	30.1% (25.2%-34.9%)	452	50.9% (45.5%-56.3%)
Trade School/College	549	31.4% (28.2%-34.6%)	1020	57.1% (53.6%-60.7%)
Bachelors or higher	714	42.9% (39.0%-46.8%)	1229	74.1% (70.6%-77.5%)
Missing		^b^	13	61.5% (36.4%-86.5%)^c^
Population Group				
White	1111	34.6% (32.2%-37.0%)	2013	60.3% (57.9%-62.8%)
Black	67	40.4% (28.9%-51.8%)	110	69.7% (58.7%-80.6%)
East/Southeast Asian	124	43.0% (35.1%-50.9%)	203	66.0% (58.2%-73.9%)
South Asian	72	35.4% (23.0%-47.8%)^c^	110	61.4% (47.2%-75.6%)
Middle Eastern	29	24.9% (13.6%-36.1%)^c^	64	68.1% (53.2%-82.9%)
Latino	27	42.0% (24.0%-60.0%)^c^	55	72.2% (53.8%-90.6%)
Indigenous	122	26.7% (19.4%-34.0%)	215	51.2% (43.3%-59.1%)
Missing	65	45.6% (32.9%-58.3%)	115	78.7% (69.1%-88.3%)
Immigrant Status				
Non-immigrant	1188	32.9% (30.5%-35.3%)	2160	59.0% (56.6%-61.4%)
5 years or less	137	42.5% (32.6%-52.4%)	214	65.4% (55.8%-74.9%)
6 to 10 years	123	45.3% (36.6%-54.1%)	196	75.9% (68.2%-83.6%)
Greater than 10 years	112	35.0% (26.5%-43.5%)	217	63.1% (54.1%-72.1%)
Missing	57	45.5% (31.9%-59.1%)^c^	98	78.0% (68.6%-87.3%)
Residence				
Rural area	385	30.8% (27.1%-34.5%)	694	53.7% (49.6%-57.8%)
Population Centre	1232	36.6% (33.9%-39.2%)	2191	63.9% (61.4%-66.4%)
Marital Status				
Married	1153	42.3% (39.4%-45.3%)	1943	70.4% (67.6%-73.2%)
Living Common Law	246	24.8% (20.8%-28.8%)	527	50.2% (45.8%-54.5%)
Widowed, Divorced, Separated	86	27.5% (20.4%-34.6%)	160	56.4% (47.8%-65.1%)
Single, Never married	132	17.5% (12.6%-22.5%)	255	36.6% (30.3%-42.9%)
Perceived Mental Health				
Poor	15	17.0% (6.5%-27.4%)^c^	31	36.8% (21.5%-52.1%)^c^
Fair	93	28.7% (21.3%-36.1%)	159	49.7% (41.3%-58.2%)
Good	389	36.4% (31.8%-41.1%)	714	62.1% (57.7%-66.5%)
Very Good	616	35.2% (31.5%-38.9%)	1131	64.4% (60.9%-67.9%)
Excellent	503	37.2% (33.1%-41.3%)	849	62.8% (58.6%-67.0%)
Missing^b^				
Pre-Pregnancy BMI (kg/m^2)^				
Underweight (< 18.5 kg/m^2^)	73	34.7% (25.4%-44.0%)	128	56.6% (46.5%-66.7%)
Normal (18.5–24.9 kg/m^2^)	882	39.4% (36.1%-42.7%)	1555	67.8% (64.8%-70.8%)
Overweight (25.0–29.9 kg/m^2^)	301	29.8% (25.4%-34.2%)	554	58.0% (53.1%-62.9%)
Obese (> 30 kg/m^2^)	162	25.6% (20.2%-31.0%)	306	47.5% (40.8%-54.3%)
Missing	199	40.5% (33.9%-47.2%)	342	64.4% (57.6%-71.2%)
Co-sleep				
Daily	699	45.6% (41.4%-49.9%)	1159	73.4% (69.6%-77.1%)
Occasional	419	33.1% (28.7%-37.4%)	786	62.6% (58.3%-66.9%)
Never	483	27.4% (24.4%-30.5%)	918	50.6% (47.3%-53.9%)
Missing	16	51.8% (22.1%-81.4%)^c^	22	88.5% (75.5%-100%)
Smoking During Last three months of Pregnancy				
Any Smoking	70	15.3% (9.7%-20.8%)^c^	147	29.2% (22.7%-35.7%)
Never	1547	37.3% (34.9%-39.6%)^c^	2734	64.8% (62.5%-67.2%)
Missing^b^				

For pre-pregnancy BMI although the Canadian guidelines are conventionally applied to individuals aged 18 and older, we applied them to five 17 year old respondents in our cohort. The CCHS defined co-sleeping as the baby and parent(s) sharing the same bed

*N* = Number of females reporting exclusive or any breastfeeding, Total cohort for any breastfeeding is 4,845 females and total cohort for exclusive breastfeeding is 4,741 females

^a^ Data are presented as proportions (95% Confidence Intervals) unless otherwise specified

^b^ Estimate has a coefficient of variation above 35 or sample size below 10

^c^ Estimate has a coefficient of variation above 15 (interpret with caution)

### Analysis

We calculated breastfeeding rates overall and across maternal characteristics. The cumulative proportion of females introducing liquids and solids, and the reasons for stopping breastfeeding were examined by the baby’s age in months. Any statistic based on less than 10 individuals or which had a coefficient of variation of more than 35% was suppressed as per Statistics Canada guidelines [11]. All analyses were carried out using sampling weights. We calculated 95% confidence intervals using the bootstrap method [11].

Using a multivariable log binomial model, we calculated adjusted prevalence ratios (aPR) for exclusive and any breastfeeding for studied maternal characteristics. We purposefully included education, province/territory of residence and population group in the initial models, as these variables have been consistently shown to influence breastfeeding behaviour [8]. For other covariates, the significance level to enter the model was set at 20% and the level to stay in the model was set at 5%. The variance inflation factor was below 1.2 for all variables, so no adjustment for multicollinearity was required. With the exception of age, all variables were treated as categorical. Reference categories were those with either the highest or lowest unadjusted breastfeeding rates with two exceptions. For province or territory of residence, British Columbia was chosen as it had high breastfeeding rates and a large population and for population group, white was chosen as the majority of the sample was white. Missing values were handled by creating a “missing” category, as missingness was spread throughout the sample and exclusion of records with missing values would have resulted in the loss of 75% of our sample. With the exception of 14.4% missing for pre-pregnancy BMI, less than 5% was missing for other variables. All analyses were conducted using SAS Enterprise Guide version 7.1.

## Results

### Exclusive breastfeeding at six months

Overall 35.6% of females (95% CI: 33.3%-37.8%) exclusively breastfed for at least six months (Table 1). Rates generally increased with maternal age and education, with the highest rates reported by females aged 40–44 and among those with a university degree. Geographically, rates were highest in the Yukon and British Columbia and lowest in Newfoundland and Labrador and Quebec. Females in urban centres reported higher six-month exclusive breastfeeding rates than females in rural areas. Immigrant females reported higher rates than non-immigrants, and across ethnic groups, East/Southeast Asian females reported the highest rates and Middle Eastern females reported the lowest. Married females had a higher prevalence than unmarried females. Additionally, six-month exclusive breastfeeding rates generally increased as perceived mental health increased, and were higher among those reporting a normal pre-pregnancy BMI compared to other BMI groups, among non-smokers in the last three months of pregnancy compared to smokers, and among females who co-slept daily with their infant compared to females who occasionally or never co-slept.

### Any breastfeeding at six months

Overall 62.2% of females (95% CI: 60.0%-64.4%) breastfed for at least six months (Table 1). The pattern of any breastfeeding across maternal characteristics was identical to that observed for exclusive breastfeeding, with the exception of population group where the highest rates were observed among Latino females and the lowest among Indigenous females.

### Timing of breastfeeding cessation

**Fig. 1:**
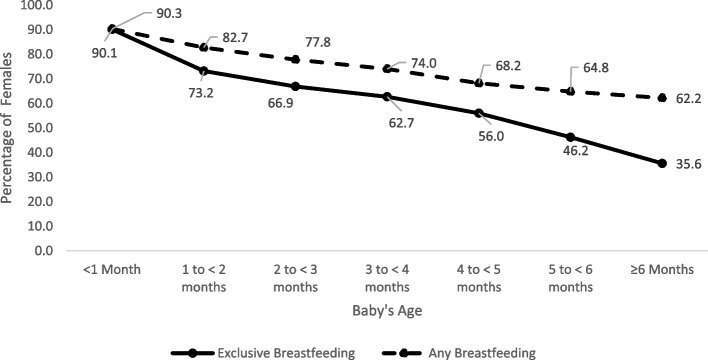
Rates of exclusive and any breastfeeding,^*^ by baby’s age, 2017–2018 Canadian Community Health Survey Legend: ^*^These rates are based on all females (those who initiated breastfeeding and those who did not breastfeed). Rates in Fig. 2 do not align completely with these, as those are based only on females who initiated breastfeeding

**Fig. 2:**
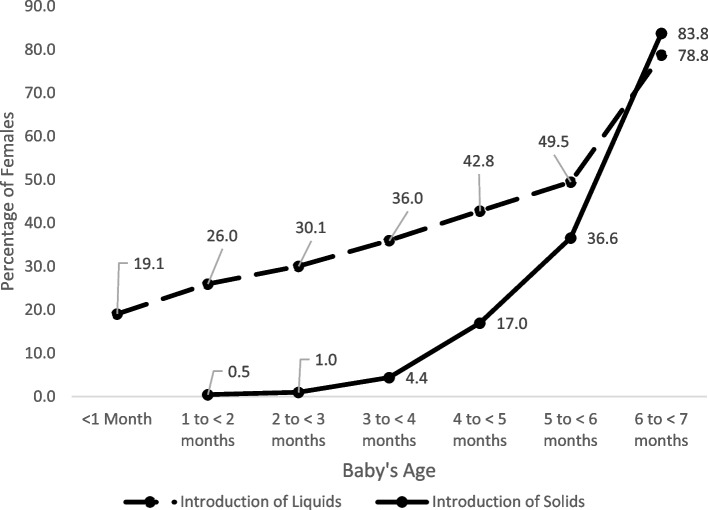
Cumulative proportion of females introducing other liquids or solid foods^*^, by baby’s age, 2017–2018 Canadian Community Health Survey Legend: ^*^These rates are based only on females who initiated breastfeeding. Rates in Fig. 1 do not align completely with these, as those are based on all females (those who initiated breastfeeding and those who did not breastfeed)

Although over 90% of females initiated exclusive breastfeeding, there was a steep decline of 20.1% in the first month (Fig. 1). After four months, the decline accelerated again with a drop of 9.8% and 10.6% prior to the ages of five months and six months, respectively. For any breastfeeding, although the steepest drop also occurred during the first month (7.6%), month-to-month rates of decline were lower than those observed for exclusive breastfeeding particulary between ages four to six months. By five months, less than half of females were exclusively breastfeeding (46.2%, 95% CI: 44.8%-47.7%), dropping to the previously mentioned 35.6% by six months. Figure 2 illustrates that the sharp decline in exclusive breastfeeding in the first month was predominantly due to 19.1% (95% CI: 16.7% to 21.5%) of breastfeeding females introducing other liquids into their child’s diet, and the accelerated decline observed after four months was predominantly due to the introduction of solid foods – 36.6% (95% CI: 34.6% to 38.7%) of breastfeeding females introduced solid foods before six months.

### Reason for stopping breastfeeding

Insufficient breast milk was the most frequent reason for stopping breastfeeding throughout the first six months, cited most often by females who stopped breastfeeding between four and five months (52.6%, 95% CI: 41.5%-63.7%) (Table 2). Difficulty with breastfeeding was the second most frequent reason; it was cited most often by females who stopped breastfeeding prior to one month (24.6%, 95% CI: 17.7%-31.4%). The third most cited reason for breastfeeding cessation was a medical condition with the mother or the baby. Other reasons – ready for solids, fatigue due to breastfeeding, planned to stop at this time, child weaned him/herself, returning to school or work – were given less frequently.

**Table 2:** Reasons females stopped breastfeeding, by baby’s age. 2017–2018 Canadian Community Health Survey

Baby’s age	Not enough breast milk	Difficulty with breastfeeding	Medical condition mother/baby	Other
**< 1 months**	39.4% (31.4%-47.5%)	24.6% (17.7%-31.4%)	15.7% (10.8%-20.6%)	20.4% (14.4%-26.2%)
**1 to < 2 months**	46.7% (36.8%-56.6%)	15.3% (9.4%-21.3%)	16.8% (9.5%-24.0%)	21.2% (12.7%-29.6%)
**2 to < 3 months**	40.2% (29.5%-50.8%)	15.8% (7.0%-24.5%)	13.8% (4.5%-23.0%)	30.2% (19.9%-40.8%)
**3 to < 4 months**	45.2% (35.4%-55.1%)	11.1% (6.7%-15.5%)	11.5% (6.5%-16.5%)	32.2% (22.4%-41.9%)
**4 to < 5 months**	52.6% (41.5%-63.7%)	8.0% (3.1%-13.9%)	14.6% (6.8%-22.4%)	24.7% (15.9%-33.7%)
**5 to < 6 months**	36.8% (23.7%-49.9%)	^a^	21.2% (3.9%-38.5%)	37.5% (24.4%-50.7%)

^a^ Estimate has a coefficient of variation above 35 or sample size below 10

### Adjusted prevalence ratios of six-month breastfeeding

**Fig. 3:**
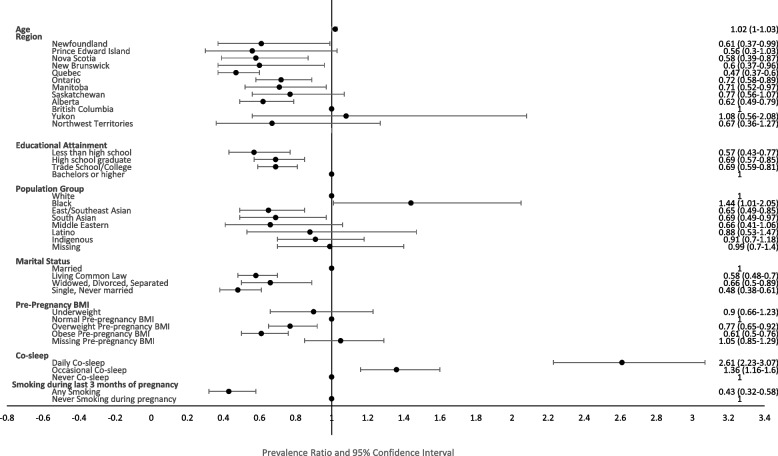
Adjusted prevalence ratios of exclusive breastfeeding for at least six months, by characteristics^*^, 2017–2018 Canadian Community Health Survey**.** Legend: * The “missing” category for those who did not report the frequency of their co-sleeping has an effect size of 3.77 (1.67–8.54) and has not been included in this graph because the confidence interval is a very wide due to this category only constituting 22 females. The missing category for smoking and education or the value for Nunavut were not shown because the estimate has a coefficient of variation above 35 or sample size below 10

Educational attainment, marital status, pre-pregnancy BMI, smoking status during the last three months of pregnancy, co-sleeping with baby and maternal age were significantly associated with six-month exclusive breastfeeding following adjustment for other characteristics, while immigrant status, perceived mental health and rural/urban status were not (Fig. 3). For significant characteristics, the direction of effect remained predominantly the same compared to unadjusted results. Higher educational attainment, being married, having a normal pre-pregnancy BMI, not smoking during the last three months of pregnancy, co-sleeping with baby and older maternal age, all increased the prevalence of six-month exclusive breastfeeding. For example, each one-year increase in age corresponded to a 2% (aPR 1.02, 95% CI: 1.0–1.03) increase in the prevalence of breastfeeding exclusively for six months. The largest aPR was observed for co-sleeping; females who co-slept daily had a 2.61 (95% CI: 2.23–3.07) times greater prevalence of exclusively breastfeeding for six months compared to those who never co-slept.

The prevalence of six-month exclusive breastfeeding also continued to vary significantly across province/territory of residence and population group, but adjusted associations differed somewhat from unadjusted results. For example, while females in the Yukon and British Columbia still had the highest prevalence; females in Newfoundland and Labrador no longer had the lowest prevalence of breastfeeding. Lower adjusted prevalence ratios were observed for Quebec, Prince Edward Island, Nova Scotia and New Brunswick. With respect to population group, Black females had the highest adjusted six-month exclusive prevalence, East/Southeast Asians and South Asian had the lowest. Notably, although regional and ethnic variation in breastfeeding prevalence persisted after adjustment, many of the aPRs were not statistically significantly different from one another (Fig. 3).

The trends in aPRs for any breastfeeding for six months showed similar patterns to those observed for exclusive breastfeeding for six months (Additional File 1).

## Discussion

Just over a third (35.6%) of females in Canada who gave birth between 2012 and 2018 met the public health recommendation to breastfeed exclusively for six months, while 62.2% did some breastfeeding for at least six months. Lower breastfeeding rates were found among females who were socially disadvantaged, such as females who were single or had lower levels of education. The largest drop in exclusive breastfeeding occurred during the first month and after the fourth month, and the most frequent reason given for breastfeeding cessation was insufficient breast milk. The following discussion focuses on exclusive breastfeeding but equally applies to any breastfeeding, as the results for any breastfeeding paralleled those for exclusive breastfeeding.

The 2012 World Health Assembly (WHA) set a target of 50% six-month exclusive breastfeeding by 2025 and 70% by 2030 [12]. Although the rate of 35.6% observed in this study is below these targets, it indicates an increase in exclusive breastfeeding from observed rates of 14.4% and 26% in 2006–2007 and 2011–2012, respectively. [6, 8]. Other countries also remain below the WHA targets, with the United States, Australia and Sweden reporting rates of 25.6%, 29% and 15% in 2017 [13–15]. As the years studied precede the COVID-19 pandemic, we were not able to study the pandemic’s impact on breastfeeding rates. Although continued breastfeeding was recommended even if COVID-19 is suspected or confirmed [16, 17], pandemic-related restrictions may have negatively impacted the amount of breastfeeding support available to females [18]. Future work to assess if and how the pandemic affected Canadian breastfeeding rates is warranted.

A review of the determinants of breastfeeding practices highlighted that successful protection, promotion and support of breastfeeding is influenced at a structural level by sociocultural and market contexts; at a settings level by health services, family and communities, and work environments; and at the level of the individual [19]. We did not study this broad range of determinants, but our findings that higher educational attainment, being married, living in certain provinces/territories and being of a particular population group, increase the prevalence of breastfeeding mirror those found in other studies [3, 8, 20, 21] and reinforce the importance of sociocultural contexts in influencing breastfeeding practices. Despite having similar findings to past studies it is important to note these trends persist. Normal pre-pregnancy BMI, which may reflect dietary patterns, and not smoking during pregnancy were also associated with increased breastfeeding. Such individual-level factors are also known to be significantly influenced by social and economic conditions throughout the life course [22]. Collectively, these sociocultural and socioeconomically influenced factors point to the need to identify and remove structural barriers that impede breastfeeding. In particular, addressing lower breastfeeding rates among socially disadvantaged females can contribute to reducing a cycle of disadvantage, as these females and their children experience the considerable benefits of breastfeeding.

At the settings level, breastfeeding practices are influenced by factors such as employment conditions and health services [19]. During the years covered by this study, Canada’s maternity/parental leave policy provided females employment-protected leave for up to one year following the birth of their child, paid at 55% or higher of pre-leave earnings [23]. Although this policy is not fully inclusive, as eligibility requirements and low rates of pre-leave pay effectively exclude some females, it nevertheless may have contributed to our finding that return to work was not one of the main reasons for cessation of breastfeeding. Canada, however, fairs less favourably with regard to the Baby Friendly Hospital Initiative (BFHI), referred to in Canada as the Baby Friendly Initiative (BFI) [24]. The BFI comprises 10 health facility-based interventions to protect, promote and support breastfeeding, with substantial evidence that they collectively improve exclusive breastfeeding [25]. In 2017, only 4.7% of births in Canada occurred in a BFI-designated health facility [26] though almost all births (97.9%) in Canada occur in a hospital [27]. The WHO recommends that countries scale up BFI implementation to universal coverage and ensure sustainability, as one strategy to increase breastfeeding exclusivity and duration [28].

Our study touched on three elements of the BFI: enabling females and infants to remain together, having access to ongoing support, and supporting females to manage common difficulties [24]. With regard to females and infants remaining together, among the variables studied, co-sleeping had the strongest association with six-month breastfeeding. Females who co-slept daily had a 2.6 times greater adjusted prevalence of exclusive breastfeeding for six months compared to females who never co-slept. There has been strong messaging against co-sleeping following studies that showed an increased risk of injury to the infant or Sudden Infant Death Syndrome (SIDS) [29]. However, messaging is now shifting towards informing parents on how to arrange a safe sleep environment in both co-sleeping and non co-sleeping environments [29, 30]. 

The largest declines in exclusive breastfeeding occurred in the first month and after four months, and the most common reasons for cessation were insufficient milk supply and difficulty with breastfeeding. These findings are similar to those of other studies [8, 10, 20, 21], and emphasize the need for early and continued postpartum breastfeeding support. Although over 50% of females in our and other studies [10] perceive insufficiency in their milk supply, biologically less than five percent of females are unable to produce adequate milk to meet the nutritional needs of their infant [7]. As an unintended consequence, introducing other liquids or solids can interrupt breast milk production [8]. Early and ongoing access to skilled breastfeeding support (e.g., lactation consultants) and peer-supports (e.g. community-based breastfeeding programs) can assist females in addressing perceptions of insufficient milk and other breastfeeding difficulties thereby increasing breastfeeding exclusivity and duration [20, 31]. The decline in exclusive breastfeeding after four months suggest this is another important time to reassert that breastmilk alone meets (most) babies’ nutritional needs up to six months of age. There is no evidence that introducing foods other than breastmilk prior to six months improves infant health [32].

Improving exclusive breastfeeding rates not only requires interventions that support females but also data systems for monitoring breastfeeding trends and assessing the impact of interventions. The CCHS provides a national picture but in-depth assessment of local barriers and facilitators to breastfeeding are also needed. For example, our results suggest that breastfeeding may be more of a social norm in British Columbia and the Yukon than in other parts of the country. Investigating the factors that contribute to this could inform breastfeeding promotion in other jurisdictions, noting that interventions must be adapted to the local context. Routine well-baby visits, which include discussion about children’s eating habits and nutritional needs, occur at 2, 4, 6, 9, 12 and 18 months, and at 2 years. These visits could serve as a source of data on breastfeeding as well as an opportunity to encourage exclusive breastfeeding until six months.

### Limitations of study

Many maternal characteristics were measured at the time of the survey (2017–2018) which could potentially be five years after the birth. Our analysis implicitly assumes that these characteristics reflect the female’s characteristics at the time of the index birth which may not be the case. For example, perceived mental health at the time of the survey (which was found not to be significantly associated with six-month breastfeeding) may not reflect postpartum mental health which has been found to influence breastfeeding behaviour [33]. Additionally, due to the self-reported nature of the survey, reports of breastfeeding experiences may be subject to recall bias and social desirability bias. The CCHS also excludes select groups such as those living in institutions or living on Indigenous reserves. We cannot assume our estimates extend to excluded subgroups; however these exclusions only account for 2% of the Canadian population 12 and over. CCHS also does not capture the breastfeeding experiences of people who do not identify as female. Due to the cross-sectional nature of the survey temporality cannot be determined. As our study used 2017–2018 data, we were unable to assess the impact of the COVID-19 pandemic on breastfeeding rates. Finally, CCHS data facilitated the study of only a limited number of maternal characteristics that could influence breastfeeding behaviour. Not being able to account for other characteristics such as parity, postpartum mental health or receipt of breastfeeding support at birth, makes our results subject to residual confounding from unmeasured factors. Despite these limitations, the nationally representative nature of the CCHS and its inclusion of questions on breastfeeding make it a valuable source of data for monitoring trends in the duration of breastfeeding in Canada and for studying some of the factors associated with breastfeeding cessation.

## Conclusion

Although Canadian exclusive breastfeeding rates are rising, the majority of females still do not meet the recommendation to exclusively breastfeed for at least six months. Given that the largest decline in exclusive breastfeeding occurs before infants are a month old, and in light of the fact that numerous societal and maternal characteristics are associated with breastfeeding duration, there continues to be a need for early and multipronged interventions to support females to exclusively breastfeed longer.

## Supplementary Information

**Additional file 1.** Adjusted prevalence ratios of any breastfeeding for at least six months, by maternal characteristics, 2017-2018 Canadian Community Health Survey *

## Data Availability

The data that support the findings of this study are available from Statistics Canada, but restrictions apply to the availability of these data, which are provided to the Public Health Agency of Canada under its national health surveillance mandate, and so are not publicly available. Data are however available from the corresponding author upon reasonable request and with the permission of Statistics Canada.

## Abbreviations

CCHS: Canadian Community Health Survey
WHO: World Health Organization
BMI: Body Mass Index
aPR: Adjusted prevalence ratios
WHA: World Health Assembly
CI: Confidence interval
BFHI: Baby friendly hospital initiative
BFI: Baby friendly initiative
SIDS: Sudden infant death syndrome

## References

[1] Kramer MS, Kakuma R. Optimal duration of exclusive breastfeeding [Internet]. The Cochrane database of systematic reviews. U.S. National Library of Medicine; 2012. Available from: https://pubmed.ncbi.nlm.nih.gov/22895934/. [Cited 26 Oct 2022]. 10.1002/14651858.CD003517.pub2 PMC7154583 22895934

[2] Khan J, Vesel L, Bahl R, Martines JC (2015). Timing of Breastfeeding Initiation and Exclusivity of Breastfeeding During the First Month of Life: Effects on Neonatal Mortality and Morbidity—A Systematic Review and Meta-analysis. Matern Child Health J.

[3] Victora CG, Bahl R, Barros AJD, França GVA, Horton S, Krasevec J (2016). Breastfeeding in the 21st century: Epidemiology, mechanisms, and lifelong effect. Lancet.

[4] Health Canada, Canadian Paediatric Society, Dietitians of Canada, Breastfeeding Committee for Canada. Nutrition for healthy term infants: recommendations from birth to six months. Can J Diet Pract Res. 2012;73(4):204. 10.3148/73.4.2012.204 23217450

[5] Government of Canada. A joint statement of Health Canada, Canadian Paediatric Society, Dietitians of Canada, and Breastfeeding Committee for Canada [Internet]. Nutrition for Healthy Term Infants: Recommendations from Birth to Six Months - Canada.ca. Gouvernement of Canada; 2022. Available from: https://www.canada.ca/en/health-canada/services/canada-food-guide/resources/infant-feeding/nutrition-healthy-term-infants-recommendations-birth-six-months.html. [Cited 26 Oct 2022] 10.3148/73.4.2012.204 23217450

[6] Public Health Agency of Canada. What Mothers Say : The Canadian Maternity. 2009.

[7] Chalmers B (2013). Breastfeeding unfriendly in Canada?. CMAJ.

[8] Gionet L. Health at a Glance - Breastfeeding trends in Canada. 2013;(82):1–7.

[9] Francis J, Mildon A, Stewart S, Underhill B, Tarasuk V, Di Ruggiero E (2020). Vulnerable mothers’ experiences breastfeeding with an enhanced community lactation support program. Matern Child Nutr.

[10] M. Jessri , AP. Farmer, K. Maximova, N.D. Willows RB. Predictors of exclusive breastfeeding: Observations from the Alberta pregnancy outcomes and nutrition (APrON) study. BMC Pediatr. 2013;13(1). Available from: http://www.embase.com/search/results?subaction=viewrecord&from=export&id=L368974974%5; http://www.biomedcentral.com/1471–2431/13/77%5; http://dx.doi.org/10.1186/1471-2431-13-77 10.1186/1471-2431-13-77 PMC3660294 23679578

[11] Canadian Community Health Survey - Annual Component (CCHS) 2017–2018. CCHS 2017–2018 User Guide. Statistics Canada; 2020. Available from: https://abacus.library.ubc.ca/file.xhtml?persistentId=hdl%3A11272.1%2FAB2%2FSEB16A%2F1SHX8R&version=1.0. [Cited 26 Oct 2022]

[12] Neves PAR, Vaz JS, Maia FS, Baker P, Gatica-Domínguez G, Piwoz E (2021). Rates and time trends in the consumption of breastmilk, formula, and animal milk by children younger than 2 years from 2000 to 2019: analysis of 113 countries. Lancet Child Adolesc Heal.

[13] Official Statistics of Sweden . Statistics on breastfeeding 2017 [Internet]. The National Board of Health and Welfare. Official Statistics of Sweden; 2019. Available from: https://www.socialstyrelsen.se/globalassets/sharepoint-dokument/artikelkatalog/statistik/2019-9-6379.pdf. [Cited 26 Oct 2022].

[14] Breastfeeding, 2017–18 financial year. Australian Bureau of Statistics. Australian Bureau of Statistics; 2018. Available from: https://www.abs.gov.au/statistics/health/health-conditions-and-risks/breastfeeding/2017-18. [Cited 26 Oct 2022].

[15] Division of Nutrition, Physical Activity and Obesity. Breastfeeding Report Card United States, 2020. Breastfeed Rep Card United States, 2020. 2020;(37):6. Available from: https://www.cdc.gov/breastfeeding/pdf/2020-Breastfeeding-Report-Card-H.pdf

[16] Canadian Paediatric Society. [Internet]. Breastfeeding and Covid-19. Canadian Paediatric Society; 2021. Available from: https://cps.ca/documents/position/breastfeeding-when-mothers-have-suspected-or-proven-covid-19. [Cited 29 Dec 2022].

[17] Breastfeeding Committee for Canada. Infant Feeding and COVID-19. Breastfeeding Committee for Canada; 2021. Available from: https://breastfeedingcanada.ca/wp-content/uploads/2021/01/BCC-Covid-19-and-Infant-Feeding-Key-Messages-Jan-6-2021-clean-copy.pdf. [Cited 29 Dec 2022].

[18] Turner S, McGann B, Brockway M’M. A review of the disruption of breastfeeding supports in response to the COVID-19 pandemic in five Western countries and applications for Clinical Practice - International Breastfeeding Journal [Internet]. BioMed Central. BioMed Central; 2022. Available from: https://internationalbreastfeedingjournal.biomedcentral.com/articles/10.1186/s13006-022-00478-5[Cited 29 Dec 2022]. 10.1186/s13006-022-00478-5 PMC9107585 35570307

[19] Rollins NC, Bhandari N, Hajeebhoy N, Horton S, Lutter CK, Martines JC, et al. Why invest, and what it will take to improve breastfeeding practices? Lancet [Internet]. 2016;387(10017):491–504. Available from: 10.1016/S0140-6736(15)01044-2 26869576

[20] Brown CRL, Dodds L, Legge A, Bryanton J, Semenic S (2014). Factors influencing the reasons why mothers stop breastfeeding. Can J Public Heal.

[21] Al-Sahab B, Lanes A, Feldman M, Tamim H (2010). Prevalence and predictors of 6-month exclusive breastfeeding among Canadian women: A national survey. BMC Pediatr.

[22] Lynch J (1997). Why Poor People Behave Poorly? Variation in Adult Health Behaviours and Psychosocial Characteristics By Stages of the Socioecomic Lifecourse. Soc Sci Med.

[23] Chzhen Y, Gromada A, Rees G (2019). Are The World’s Richest Countries Family-Friendly? Policy in the OECD and EU. UNICEF Off Res.

[24] Pound CM, Unger SL (2012). The baby-friendly Initiative: Protecting, promoting and supporting breastfeeding - Nutrition and Gastroenterology Committee and Hospital Paediatrics Section Canadian Paediatric Society. Paediatr Child Heal.

[25] Breastfeeding Committee for Canada. Who we are. Breastfeeding Committee for Canada. Breastfeeding Committee for Canada; 2022. Available from: https://breastfeedingcanada.ca/en/who-we-are/. [Cited 26 Oct 2022].

[26] Dumas, Louise;Venter K. The Baby-Friendly Initiative (BFI) in Canada: Status Report 2017. 2017;1–34. Available from: http://breastfeedingcanada.ca/documents/BFI_Status_report_2012_FINAL.pdf

[27] Statistics Canada. Live births and fetal deaths (stillbirths), by place of birth (hospital or non-hospital). Statistics Canada; 2022. Available from: https://www150.statcan.gc.ca/t1/tbl1/en/tv.action?pid=1310042901. [Cited 26 Oct 2022].

[28] World Health Organization. Promoting baby-friendly hospitals. World Health Organization; [cited 2022Oct26]. Available from: https://www.who.int/activities/promoting-baby-friendly-hospitals. [Cited 26 Oct 2022].

[29] Blair PS, Ball HL, McKenna JJ, Feldman-Winter L, Marinelli KA, Bartick MC (2020). Bedsharing and Breastfeeding: The Academy of Breastfeeding Medicine Protocol #6, Revision 2019. Breastfeed Med.

[30] Safe Sleep for Your Baby. Public Health Agency of Canada. Government of Canada; 2022. Available from: https://www.canada.ca/en/public-health/services/health-promotion/childhood-adolescence/stages-childhood/infancy-birth-two-years/safe-sleep/safe-sleep-your-baby-brochure.html#a1. [Cited 26 Oct 2022].

[31] McFadden A, Gavine A, Renfrew MJ, Wade A, Buchanan P, Taylor JL, et al. Support for healthy breastfeeding mothers with healthy term babies. Cochrane Database Syst Rev. 2017 Feb 28;2017(2). Available from: http://doi.wiley.com/10.1002/14651858.CD001141.pub5 10.1002/14651858.CD001141.pub5 PMC6464485 28244064

[32] Smith HA, Becker GE. Early additional food and fluids for healthy breastfed full-term infants. Cochrane Database Syst Rev. 2016 Aug 30;2016(8). Available from: http://doi.wiley.com/10.1002/14651858.CD006462.pub430. 10.1002/14651858.CD006462.pub4 PMC8588276 27574798

[33] Pope CJ (2016). Mazmanian D.

